# No Experimental Evidence of Co-Feeding Transmission of African Swine Fever Virus between *Ornithodoros* Soft Ticks

**DOI:** 10.3390/pathogens9030168

**Published:** 2020-02-28

**Authors:** Rémi Pereira De Oliveira, Evelyne Hutet, Maxime Duhayon, Frédéric Paboeuf, Marie-Frédérique Le Potier, Laurence Vial

**Affiliations:** 1 UMR ASTRE, CIRAD, Campus International de Baillarguet, F-34398 Montpellier, France; remi.pereira_de_oliveira@cirad.fr (R.P.D.O.); maxime.duhayon@cirad.fr (M.D.); 2 UMR ASTRE, Univ Montpellier, CIRAD, INRAE, Campus International de Baillarguet, F-34398 Montpellier, France; 3 Swine Virology and Immunology Unit, Ploufragan-Plouzané-Niort Laboratory, ANSES, 35 rue de Beaucemaine, 22440 Ploufragan, France; Evelyne.HUTET@anses.fr (E.H.); Frederic.PABOEUF@anses.fr (F.P.); marie-frederique.lepotier@anses.fr (M.-F.L.P.)

**Keywords:** African swine fever, Argasid tick, co-feeding, *Ornithodoros moubata*

## Abstract

*Ornithodoros* soft ticks are the only known vector and reservoir of the African swine fever virus, a major lethal infectious disease of *Suidae*. The co-feeding event for virus transmission and maintenance among soft tick populations has been poorly documented. We infected *Ornithodoros moubata*, a known tick vector in Africa, with an African swine fever virus strain originated in Africa, to test its ability to infect *O. moubata* through co-feeding on domestic pigs. In our experimental conditions, tick-to-tick virus transmission through co-feeding failed, although pigs became infected through the infectious tick bite.

## 1. Introduction

African swine fever (ASF) is a lethal disease of *Suidae* caused by the African swine fever virus (ASFV). ASF is endemic in southern and eastern Africa, where ASFV circulates among different hosts, such as domestic pigs and warthogs, as well as soft tick vectors from the *Ornithodoros moubata* group [1,2,3,4]. Three main transmission cycles are commonly described in these endemic areas: (i) the sylvatic cycle, corresponding to ASFV transmission between warthogs and ticks, (ii) the domestic/tick cycle, corresponding to ASFV transmission between domestic pigs and ticks, and (iii) the domestic cycle, corresponding to ASFV circulation between domestic pigs only [5,6]. It is assumed that soft ticks play an important role in maintaining ASFV within the sylvatic cycle, as well as in transmitting ASFV between wild and domestic *Suidae* [4,7,8].

To be infected, ticks need to engorge on viremic animals with a sufficient viral load [9]. As viremia in warthogs is typically low (or undetectable), Plowright (1977) assumed that viremia in warthogs could not be sufficient to infect soft ticks [10]. He concluded that warthogs may only act as carriers of infected soft ticks, thanks to their movements between wild and domestic areas [10]. Conversely, Thomson (1980) conducted experimental infections of young warthogs, that are most commonly reported to be infected in the wild, and also the only stage that remains in close contact with endophilous soft ticks in burrows [11]. Against all expectations, those warthogs developed high viremia, at least for two weeks, which was considered sufficient to infect soft ticks. The use of new-born warthogs might explain the higher virus titers observed because of the immunological immaturity of the animals [11]. To date, the role of the sylvatic cycle between warthogs and soft ticks in maintaining ASFV in African wild areas has remained unclear. At the same time, other studies have demonstrated that soft ticks can also become infected without vertebrate hosts through ASFV vertical transmission to descendants [9,12] and ASFV sexual transmission between males and females during copulation [13], at least with some tick species and viral strains.

Apart from such tick-to-tick transmission modes, co-feeding might also be assumed to be an alternative or complementary route of ASFV transmission to sexual and vertical transmission in this specific context. Indeed, co-feeding is described as an efficient non-systematic transmission pathway allowing tick infection through biting on hosts with low or no viremia. It occurs when infected and uninfected ticks take their blood meal in spatio-temporal proximity on the same non-viremic [14,15] or immunized vertebrate host [16]. This mode of transmission has been demonstrated in hard ticks for certain viruses, such as the Thogoto virus [17], the Tick-borne encephalitis virus [18], the Crimean-Congo hemorrhagic fever virus [19], and for bacteria such as *Borrelia afzelli* [20]. To our knowledge, only one study conducted by Rennie (1998) has evaluated co-feeding as another pathway for ASFV dissemination among soft tick populations. However, the results were not conclusive [21].

In our study, we experimentally assess the ASFV transmission through co-feeding, using infected and uninfected *O. moubata* ticks and an ASFV strain originally isolated from the same tick species in the wild [22]. This study is part of investigations testing different pathways of ASFV transmission by soft ticks.

## 2. Materials and Methods

The animal experiments were authorized by the French Ministry for Research (project No. 2017062615498464) and approved by the national ethics committee (authorization No. 11/07/17-3).

The Liv13/33, a hemadsorbing ASFV strain (genotype I), used for tick infections was isolated from *O. moubata* in Livingstone park, Zambia in 1983 [22] and was kindly provided by Dr. Linda Dixon (OIE reference laboratory, Pirbright Institute, UK).

The *O. moubata* ticks (“Neuchâtel strain”) used in the experiment were *O. moubata* sensu stricto, as described by Bakkes et al. (2018) [1]. They originated from Southern Africa and were maintained in the Neuchâtel University insectary for at least twenty years, and then reared at CIRAD since 2008.

Males and females of *O. moubata* were infected with the Liv13/33 strain by blood feeding on viremic pigs with a viremia ranging from 10^7,8^ hemadsorbing dose 50%/mL (HAD_50_/mL) to 10^8,1^HAD_50_/mL, as previously described [23]. The resulting *O. moubata*- Liv13/33 tick-virus pair (OmL) was used as the “source” for the co-feeding transmission trial 8 months post infection. OmL was previously confirmed to be competent to transmit ASFV to pigs through biting, 2 and 8 months post infection [24]. Furthermore, all OmL tested by real-time PCR were highly positive for ASFV [24].

Uninfected *O. moubata* ticks (OmCF), at the first nymphal development stage (nymphs-1), were used as “recipient” for the co-feeding transmission trial. Nymphs-1 were preferred, so as to be easily distinguished after engorgement from the infected “source” adult ticks.

The co-feeding transmission trial was conducted using two replicates: 1) eight infected females and seven infected males mixed with 30 uninfected nymphs-1 (group 1), and 2) nine infected females and six infected males mixed with 30 uninfected nymphs-1 (group 2). Each of the two groups were fed at the same time on the same specific pathogen-free (SPF) pig (7 weeks-old Large White female) in the same Petri dish (6 cm diameter) for 3 hours. Petri dishes were fixed to the pig’s abdomen with a bandage. The abdomen zone was chosen because the skin is thin, which eases the engorgement of ticks.

After engorgement, the ticks were washed to prevent external contamination and then sorted into “engorged” versus “non-engorged” ticks, distinguishing between adults and nymphs-1 (Table 1). Ticks’ feces and coxal fluid, excreted and secreted respectively during the tick blood meal, were collected from the bottom of both Petri dishes containing the two tick groups, to explore also the possibility for nymphs-1 to become infected by direct contact with these excretions and/or secretions.

As a positive control to ensure the ability of *O. moubata* nymphs-1 to become infected through the blood meal, a third group of uninfected *O. moubata* nymphs-1 (OmCTR), containing 11 specimen of OmCTR ticks were engorged on the same experimental pig when it became viremic (with a viremia of 10^7.8^ HAD_50_/mL) after the engorgement of infected ticks with the Liv13/33 strain.

A specific real-time polymerase chain reaction (PCR) for the detection of the ASFV VP72 gene [25] and beta-actin of ticks was used on individual DNA extracts from both tick samples, OmCF and OmCTR, two months after the co-feeding trial, as previously described [24]. Tick excretions and/or secretions collected from the Petri dishes were also tested for viral genome detection. The results were expressed as the cycle threshold (Ct) for a qualitative analysis, to define the positive or negative status of the tick samples for ASFV.

A statistical analysis was carried out with RStudio (Version 1.1.463). A Kruskal test was used to compare the amplification of beta-actin, a tick housekeeping gene used as an internal control for DNA extraction quality, between the three tick groups.

## 3. Results and Discussion

As a result of the engorgement of adult OmL during the co-feeding test, the pig became infected by ASFV in two days. For the ticks, no statistical difference was found for the beta-actin results between OmCTR and groups 1 and 2 of OmCF (*p*-value = 0.596), indicating that DNA extractions were homogeneous and comparable. All ticks of the OmCTR group were positive for ASFV (mean Ct = 23.62 [23.06–24.71]) (Table 1), confirming that nymphs-1 could be infected by the Liv13/33 ASFV strain through an infectious blood meal. Conversely, all OmCF ticks were negative for ASFV (No Ct) (Table 1), indicating the failure of ASFV co-feeding transmission between infected *O. moubata* adults and uninfected *O. moubata* nymphs-1. However, the ASFV genome was detected in the tick excretions and/or secretions collected from the Petri dishes of groups 1 and 2, where OmL and OmCF ticks were mixed, with real-time PCR results for ASFV detection of 28.20 Ct and 29.14 Ct for groups 1 and 2, respectively. This confirmed that adult ticks were infectious but could not infect nymphs-1 by direct contact with their infectious fluids.

The experimental design defined in the study was able to guarantee the reliability of the results obtained. Indeed, to maximize the success of transmission we used the African tick species *O. moubata,* which is known to acquire the Liv13/33 virus strain in salivary glands after infectious engorgement [21] and to transmit this virus to domestic pigs through biting [24]. To mimic as much as possible the natural conditions leading to tick infection through co-feeding [26], the transmission trial was carried out in small Petri dishes conducive to natural soft tick aggregation during the blood meal [27]. Naive nymphs-1 engorged synchronously with infected adults on uninfected pigs, as observed in natural soft tick populations.

Our results completed those obtained on pigs by Rennie (1998) [21], who suggested a failure of co-feeding transmission with the *O. moubata*-Liv13/33 tick-virus pair. However, her experimental design was quite different, since she used adult stages for both the infected “source” and the uninfected “recipient” ticks and separated them on the pig with a cardboard barrier inside the feeding unit to distinguished them after blood feeding. Interestingly, in a second trial, Rennie (1998) carried out co-feeding on mice, which is a non-sensitive animal for ASFV, and that time transmission occurred [21]. In this experiment, ticks were no longer separated but marked with a non-toxic light paint, and the only major difference from our study was the choice of the vertebrate host for blood feeding. However, Rennie attributed the successful infection to direct contact with infectious “source” ticks’ coxal fluid secreted during the blood meal, which is apparently incongruent with our results. Therefore, ASFV co-feeding transmission between ticks may depend on the nature of the vertebrate host. Indeed, events that occur directly after tick bites (immunological responses and the spread of the pathogen in the vertebrate host) may impact co-feeding success and should be further investigated. In our experimental conditions, although the pig became sick 2 days after the engorgement of the infected adult ticks, which confirmed a substantial quantity of virus injected by the ticks, it is still possible to assume that this quantity was not sufficient to infect nymphs-1 by co-feeding.

Taking our results and those of Rennie together, we can suggest that ASFV co-feeding cannot occur in domestic pigs using the *O. moubata*-Liv13/33 tick-virus pair, but additional experiments are needed to confirm these results. Co-feeding studies with others tick development stages are also needed to take into account the impact of the difference in hypostome (mouthparts) length. This difference may have an impact on the depth of the bite, and the nymphs-1 could be unable to acquire the virus secreted by the infected adults during the blood meal. Warthogs, which are susceptible to ASFV, but which develop no or low viremia when they are infected, might mediate ASFV in the same manner as domestic pigs and may not be a major route for tick infection through co-feeding in the wild. Conversely, it is not possible to exclude the role of non-susceptible animals that may colonize ancient warthog burrows where soft ticks still settle, thereby contributing to tick infection through co-feeding and thus the persistence of the virus in tick populations. ASFV maintenance in soft ticks in the field is one cause of ASFV re-emergence [28]. However, the co-feeding transmission pathway has mainly been described in hard ticks, which remain attached to their host for days for blood feeding, while soft ticks can feed in less than an hour. The duration of the blood meal might also make co-feeding transmission less likely to happen in soft tick species.

## Figures and Tables

**Table 1:** Proportions of ticks engorged during the co-feeding trial and real-time PCR results of ASFV and beta-actin detection. Results are shown as mean Ct [min Ct; max Ct]. F = female, M = male, Ct = Cycle threshold.

Groups of Ticks	Infected Adults (OmL) Number of Engorged/Total Adults	Uninfected nymphs-1 (OmCF) Number of Engorged/Total nymphs-1	Status of Pig Used for Trial	ASFV Detection in Engorged Nymphs (Ct)	Beta-actin Detection in Engorged Nymphs (Ct)
1	F: 6/8; M: 5/7	30/30	Healthy	No Ct	30,77 Ct [28.74 Ct; 32.24 Ct]
2	F: 7/9; M: 4/6	28/30		No Ct	30,09 Ct [28.96 Ct; 31.55 Ct]
Control (OmCTR)	Not present	11/11	Infected (Liv13/33 strain, 10^7.8^ HAD_50_/mL)	23,62 Ct [23.06 Ct; 24.71 Ct]	30,86 Ct [30.29 Ct; 31.5 Ct]
